# Vascular injuries during laparoscopic donor nephrectomy and proposed risk reduction strategies

**DOI:** 10.1590/S1677-5538.IBJU.2018.0281

**Published:** 2019

**Authors:** Parag Sonawane, Arvind Ganpule, Abhishek Singh, Ravindra Sabnis, Mahesh R. Desai

**Affiliations:** 1 Department of Urology, Division of Laparoscopic and Robotic Surgery, Muljibhai Patel Urological Hospital, Nadiad, Gujarat, India

## Abstract

**Introduction:**

Laparoscopic donor nephrectomy (LDN) has become the standard of care and popular among most of the transplant centres across the globe.

Objective of this video is to report different vascular injuries, their management during LDNs and propose risk reduction strategies.

**Patient and methods:**

This was a retrospective analysis of all the LDNs performed between January 2011 and March 2016. All donor nephrectomies were performed laparoscopically by transperitoneal route, under ideal operative conditions by expert laparoscopic surgeons and by novice surgeons.

**Results:**

858 LDNs (left, n = 797; right, n = 61) were performed during the study period with 5 cases of vascular injuries. Mean (SD) donor age was 45.5 (± 10.76) years and the operative time was 165 (± 44.4) min. Of these five cases, two had renal vein injury, while the three others had renal artery, inferior vena cava and aortic injury (one each). Four injuries occurred during left LDN and only one during a right LDN. Vascular injuries were managed using the Rescue stitch or metallic clips as indicated. Risk reduction strategy was developed to avoid vascular injuries during LDN, which include - meticulous attention to port placement, addition of fourth port, complete dissection of upper pole and pedicle before clipping, and judicious use of ultrasonic diathermy.

**Conclusions:**

Careful evaluation of computed tomography angiography just before surgery will act like a global positioning system (GPS) for the operating surgeon. Rescue stitch is a saviour. Not to panic and being well versed with the risk reduction strategies of laparoscopy and rescue measures is of paramount importance.

## ARTICLE INFO

**Available at: http://www.intbrazjurol.com.br/video-section/20180281_Sonawane_et_al**

**Int Braz J Urol. 2019; 45 (video #4): 193-193**

